# Anti-Inflammatory, Antinociceptive, and Antipyretic Potential of Methanol Extract of *Strychnos henningsii* in Animal Models

**DOI:** 10.1155/ijin/3982255

**Published:** 2025-11-06

**Authors:** Chrisphine Kabiro Mbugua, John K. Mwonjoria, Eliud N. M. Njagi

**Affiliations:** Department of Biochemistry, Microbiology, and Biotechnology, Kenyatta University, Nairobi, Kenya

## Abstract

Inflammation, pain, and fever cause discomfort and misery and lower the productivity and quality of life among the victims. The severe effects of synthetic drugs used to treat these conditions necessitate the need for alternative therapeutic agents. *Strychnos henningsii* is used in folkloric medicine to manage inflammation, pain, and fever, although scientific evidence to validate these claims is lacking. This study aimed to determine the in vivo anti-inflammatory, antinociceptive, and antipyretic potential of the methanol extract of *S. henningsii*. In the anti-inflammatory, antinociceptive, and antipyretic assays, animals (*n* = 5) were randomly assigned into six groups: normal control, negative control, diclofenac control, and extract-treated at 25, 50, and 100 mg/kg body weight (bw). Inflammation and pain were induced through injection of 5% formalin (50 μL) in the left hind paw, while pyrexia was induced through intraperitoneal injection of steam-distilled turpentine (20 mL/kg bw). The extract was also subjected to phytochemical screening using gas chromatography–mass spectrometry (GC-MS). The extract at the three doses significantly reduced paw edema, time spent in nociception, and rectal temperature relative to the negative control (*p* < 0.05), indicating anti-inflammatory, antinociceptive, and antipyretic effects, respectively. In the fourth hour, the extract at 25, 50, and 100 mg/kg bw inhibited paw edema by 4.34 ± 0.15, 6.13 ± 0.29, and 7.43 ± 0.42%, respectively. In the early and late phases, the extract at 100 mg/kg bw inhibited pain by 61.18 ± 0.75 and 66.71 ± 0.93%, respectively. In the 4th hour, the extract at 25, 50, and 100 mg/kg bw inhibited pyrexia by 1.97 ± 0.13, 2.39 ± 0.17, and 2.54 ± 0.17%, respectively. These effects were dose-dependent and were associated with phytochemicals identified using GC-MS analysis, such as terpenes, polyphenols, fatty acids, and salicylates. The study concluded that the extract possesses phytocompounds with anti-inflammatory, antinociceptive, and antipyretic potential and could be an alternative therapeutic agent against pain, inflammation, and pyrexia.

## 1. Introduction

Inflammation is defined as the body's reaction against cellular and tissue damage caused by trauma, noxious chemicals, burns, or bacterial, viral, and fungal infections [1]. Acute inflammation is essential for recovery and repair, but persistent inflammation can cause several ailments, such as autoimmune diseases, cancer, and cardiovascular diseases [2]. Pain is a noxious sensation and emotional experience caused by thermal, mechanical, or chemical stimuli. It acts as a warning system, protecting the body from harm [3]. Fever/pyrexia refers to an elevation in the body core temperature above the hypothalamic set point (36.6°C–37.5°C) in response to pyrogens such as interleukin-1beta, interleukin-6, and tumor necrosis factor-alpha generated by infections or as a result of inflammation, graft rejection, cancer, or autoimmune reactions [4].

Inflammation, pain, and fever are major causes of discomfort and suffering among the human populations [5–7]. Nonsteroidal anti-inflammatory drugs like diclofenac and indomethacin [8], analgesic drugs such as naproxen, diclofenac, and morphine [9], and antipyretic drugs such as acetaminophen and aspirin [4] are highly prescribed in the treatment of inflammation, pain, and pyrexia, respectively. Nevertheless, these drugs have been documented to possess undesirable effects such as nephrotoxicity, hepatotoxicity, and gastrointestinal toxicities [9, 10]. This necessitates the search for alternative medicinal agents.

Medicinal plants possess phytocompounds with various therapeutic effects in treating ailments and disorders, including inflammation, pain, and pyrexia [11–13]. These phytocompounds are considered safe, biodegradable, and more efficacious compared to synthetic drugs [14]. The plant-derived medicines are also used in modern medicine, and they are acknowledged as leads in the development of novel new drugs [15].

*Strychnos henningsii* Gilg, also known as “cape teak” or “red bitterberry,” is a tiny tree found in southern and eastern Africa. It has been established to possess therapeutic activities such as antimicrobial, anticancer, antioxidant, antiplasmodial, antimalarial, and antidiabetic activities [16]. Research studies have also identified various phytochemicals from the root, stembark, and leaf extracts of *S. henningsii*. These include monoterpene indole alkaloids (henningsamine, henningsoline, retuline, and isoretuline), phytosterols (β-sitosterol), flavonoids (catechin, epicatechin, and proanthocyanidins), and pentacyclic triterpenes (henninglupal, 19-ethylene henningsyl, and 19-ethylene henningsal). The extracts have also been reported to exert low cytotoxic effects [17].

*Strychnos henningsii* Gilg. is utilized to manage inflammation, pain, and pyrexia among communities residing in Kenya. However, the scientific evidence to support these claims is lacking. The current investigation aimed to determine the potential of methanol extract of *S. henningsii* (MESh) against pain, edema, and pyrexia in animal models.

## 2. Materials and Methods

### 2.1. Medicinal Sample Harvesting and Processing

Plant samples of *S. henningsii* were obtained from Kajiado, Kenya. The medicinal samples were collected in the dry season (January 2024) while observing bioconservation measures. The samples were botanically authenticated by a taxonomist at the Kenyatta University herbarium, and a voucher specimen (CKM 001) was deposited. The stem bark was cut into small parts for easy shade drying and milling into fine powder before extraction.

### 2.2. Sample Extraction

Approximately 500 g of the powdered sample was soaked in 2 L of methanol, vortexed for 2 h, and then left to stand for 48 h with occasional shaking. The mixture was filtered using filter paper (Whatman's No. 1) before concentration using a rotor evaporator at 64°C under reduced pressure. The resulting semisolid concentrate was put in an oven and dried at 35°C for 2 days. The solid crude extract was put in a glass bottle, sealed, and then refrigerated at 4°C. The extract percentage yield was calculated according to the formula used by Moriasi et al. [18]:

(1) $$\begin{array}{c} \%\text{ extract yield}=\frac{\text{Mass of the extract obtained}}{\text{Mass of the sample}}\times 100. \end{array}$$

### 2.3. Laboratory Animals

The acute toxicity, anti-inflammatory, and antinociceptive assays were carried out using Swiss albino mice weighing between 20 and 25 g (both sexes), while the antipyretic assays used male Wistar rats weighing 150–180 g. These animals were between 8 and 9 weeks old. The animals were acclimatized for 7 days before experimentation. The animals were kept at room temperature with equal day and night lengths. Polypropylene cages were used to house the animals throughout the study period. A standard diet from Unga Feeds was used to feed the animals, and clean water was provided *ad libitum*.

### 2.4. Acute Toxicity Assay

The mice were randomly categorized into four groups (*n* = 5): normal control (received distilled water) and three doses of the extract of 150, 548, and 2000 mg/kg bw. The extract and distilled water (0.2 mL) were administered orally once. Indicators of toxicity were monitored according to the Organization for Economic Co-operation and Development (OECD) guidelines 425. They included drug-related toxicity signs (such as over-excretion of saliva, diarrhea, tremors, change in fur alignment, change in eye colors, and lethargy), changes in behaviors (like convulsions, agitation, paralysis, unusual locomotion, and clumping together), and even mortality. The mice were monitored for 14 days [19].

### 2.5. Anti-Inflammatory Assay

The subplantar region of the left hind paw of mice was injected 5% formalin solution (50 μL) to induce inflammation, with the exception of normal control mice. The mice (*n* = 5) were randomly categorized into six groups as follows: Normal control and negative control received distilled water (vehicle); positive control mice received 15 mg/kg body weight (bw) of the reference drug, diclofenac, while the extract-treated mice were administered the extract at doses of 25, 50, and 100 mg/kg bw. The vehicle, diclofenac, and the extract were orally administered (0.2 mL) 1 h after injection of formalin [20].

The animals' paw diameters were measured using a digital Vernier caliper. The paw diameter was measured before induction of edema, 1 h after injection of formalin, and every hour for 4 h after treatment. The percentage of edema inhibition was computed based on the formula used by Mworia et al. [21] as follows:

Where;

(2) $$\begin{array}{c} \text{Percentage edema inhibition}=\frac{C-T}{C}\times 100, \end{array}$$

*C* = Paw diameter values at hour 1 after induction of edema. *T* = Paw diameter values at subsequent hours after induction of edema.

### 2.6. Antinociceptive Assay

The mice, except for the normal control mice, were injected 50 μL of 5% formalin solution in the subplantar tissue in their left hind to induce pain. Swiss albino mice were randomly assigned to six groups (*n* = 5) and then treated as follows: Normal control and negative control mice received distilled water; positive control group mice received diclofenac (15 mg/kg bw), while the extract-treated mice received the extract at 25, 50, and 100 mg/kg bw.

The vehicle, diclofenac, and the extract were orally administered (0.2 mL) 30 min before the injection of formalin. After pain induction, the animals were put in a transparent observation chamber. Indicators of pain, including lifting, licking, biting, and flinching of the left hind paw, were observed, and time spent was recorded. Nociception was recorded in two phases: early and late phases. The early phase was between 0 and 5 min, while the late phase was between 15 and 30 min after induction of pain [22]. A formula used by Kimuni et al. [23] was used to compute percentage pain inhibition as follows:

Where;

(3) $$\begin{array}{c} \text{Percentage pain inhibition}=\frac{N-T}{N}\times 100, \end{array}$$

*N* = Values of the edema control group at each phase. *T* = Values of the other treatment groups at each phase.

### 2.7. Antipyretic Assay

The male Wistar rats were induced with pyrexia using an intraperitoneal injection of steam-distilled turpentine solution (20 mL/kg bw). The experimental animals whose rectal temperature increased by 0.8 °C were considered pyretic and used in the study. The pyretic rats were randomly categorized into 5 groups (*n* = 5) and treated as follows: Negative control rats received the vehicle, positive control rats received 15 mg/kg bw of diclofenac, while the extract-treated rats were administered the extract doses of 25, 50, and 100 mg/kg bw. An additional group of normal control was also introduced. The normal control rats were not induced with pyrexia and received the vehicle. The vehicle, diclofenac, and the extract (0.5 mL) were orally administered 1 h after injection of turpentine.

A digital thermometer was inserted about 2.5 cm into the rat's rectum to measure rectal temperature before induction of pyrexia, 1 h after injection of turpentine, and every hour for 4 h after treatment [24]. The percentage of fever inhibition was calculated according to the formula used by Mworia et al. [25] as follows:

(4) $$\begin{array}{c} \text{Percentage fever inhibition}=\frac{C-T}{C}\times 100, \end{array}$$

*C* = Rectal temperature values at the first hour after induction of fever. *T* = Rectal temperature values at subsequent hours after induction of fever.

### 2.8. Gas Chromatography–Mass Spectrometry (GC-MS) Analysis

A GC-MS (7890/5975 Agilent Technologies) was utilized to analyze the extract phytochemical profile. Approximately 100 mg of the extract was dissolved in 1 mL of dichloromethane, vortexed for 10 s, sonicated for 10 min, and then centrifuged at 14,000 rpm for 5 min. The supernatant was filtered, and then a stock solution of 100 ng/μL was prepared. The extract was analyzed in full scan mode in triplicate.

A gas chromatograph of a capillary diameter of 0.25 mm and a column length of 30 m with a 0.25 μm film thickness was used. During the mass spectrophotometry phase, an electron ionization system was utilized for detection and identification. The parameters for the system were set as follows: ionization energy at 70 eV, ion source temperature of 230 °C, injection volume of the extract of 1 μL at 250 °C, helium gas flow speed of 1.25 mL/min, scan speed at 1666 microseconds, solvent cut time of 3.3 min, mass transfer line temperature of 200 °C, interface temperature at 250 °C, relative detector gain mode, a scan range of 40–550 m/z, and a runtime of 70 min.

The analysis of the standard, undecane, was also performed in full scan mode at serial dilutions ranging from 1 to 100 ng/l. A linear calibration curve was generated (*y* = 186096*x* + 1000000) and used to compute the concentration of each component in the sample. The different phytocompounds present in the methanol extract were identified by obtaining a match from the National Institute of Standards and Technology (NIST) database.

### 2.9. Statistical Analysis

Raw data were keyed into the Microsoft Excel spreadsheet and then subjected to statistical analysis using the Statistical Package for Social Sciences version 26.0. Mean and standard error of the mean (SEM) were used to express descriptive statistics. One-way analysis of variance (ANOVA) and Tukey's multiple comparisons were used to analyze for significant variations among different treatment groups. In the analysis across the treatment period, repeated measures ANOVA was used, followed by Bonferroni-adjusted multiple comparisons. The significance level was set at *p* < 0.05.

## 3. Results

### 3.1. Acute Toxicity Effect of the Extract

The mice that received a single dose of MESh at 150, 548, and 2000 mg/kg bw did not report any fatality for 14 days. The extract had a lethal dose at 50% (LD_50_) exceeding 2000 mg/kg bw in mice. In addition, the extract did not reveal any toxicity signs (such as tremors, change in fur alignment, overexcretion of saliva, diarrhea, change in eye colors, and lethargy) or behavioral changes (like agitation, paralysis, convulsions, unusual locomotion, and clumping together) after a single-dose exposure of the extract at the three doses.

### 3.2. Anti-Inflammatory Effect of the Extract

The percentage change in paw diameter in negative control mice was significantly higher relative to those in normal control, diclofenac control, and the extract-treated mice in hours 1, 2, 3, and 4 after treatment (*p* < 0.05; Table 1). The effect of the extract at 25, 50, and 100 mg/kg bw showed no significant variations in the percentage change in paw diameter in hours 1 and 2 (*p* > 0.05). However, in hour 4, the effect of the extract at 100 mg/kg bw significantly lowered paw edema relative to the dosages of 25 and 50 mg/kg bw (*p* < 0.05). The extract reduced paw edema dose-dependently in the entire experiment. The effect of the reference drug statistically matched that of the extract at the three doses on the percentage reduction in paw edema in hour 1, as well as the effect of the extract at 100 mg/kg bw in the 4th hour (*p* > 0.05; Table 1).

In the analysis across the entire experiment, no changes were noted in paw diameter of normal control mice (Table 1). There was a significant increase in the percentage change in paw diameter between hour 1 and hour 4 in negative control mice (*p* < 0.05). The mice that received diclofenac and the extract at the three doses revealed a significant reduction in paw diameter between the 1st and the 4th hours (*p* < 0.05; Table 1).

### 3.3. Antinociceptive Effect of the Extract

The indicators of pain, such as lifting, licking, biting, and flinching of the left hind paw, were recorded in the early and late phases (Table 2). There were no observable pain signs in normal control mice in both phases. The parentage pain inhibition in mice that received the extract at the dose of 100 mg/kg bw was significantly higher than those of mice that received the extract at 25 and 50 mg/kg bw at the two phases (*p* < 0.05). The extract reduced the pain dose-dependently in both phases. The percentage pain inhibition of the reference drug, diclofenac, was statistically greater than the percentage pain inhibition of the extract at the three doses in the early and late phases (*p* < 0.05). No percentage of pain inhibition was reported in negative control mice (Table 2).

### 3.4. Antipyretic Effect of the Extract

In the 1st, 2nd, 3rd, and 4th hours after treatment, the turpentine control rats had significantly higher rectal temperatures than the rectal temperatures of the other studied groups (*p* < 0.05; Table 3). In the same period, the rats in the normal control group had significantly lower rectal temperatures than those of the other tested groups (*p* < 0.05; Table 3). In hours 1, 3, and 4 after treatment, the rats that received 25, 50, and 100 mg/kg bw of the extract revealed no significant variation in the percentage of fever inhibition (*p* > 0.05). The extract reduced elevated rectal temperatures dose-dependently in the entire study (Table 4.3). The percentage of pyrexia inhibition in rats that were treated with diclofenac did not differ significantly compared to inhibition of the extract dosage of 100 mg/kg bw (*p* > 0.05) in hours 1, 2, and 3 (Table 3).

Across the treatment period, the mice in the normal control and negative control groups revealed no significant variations in the rectal temperatures (*p* > 0.05). The percentage of fever inhibition significantly increased between hour 1 and hour 4 in rats that were administered diclofenac and the extract at the three doses (*p* < 0.05; Table 3).

### 3.5. Phytochemical Profile of the Extract

**Figure 1:**
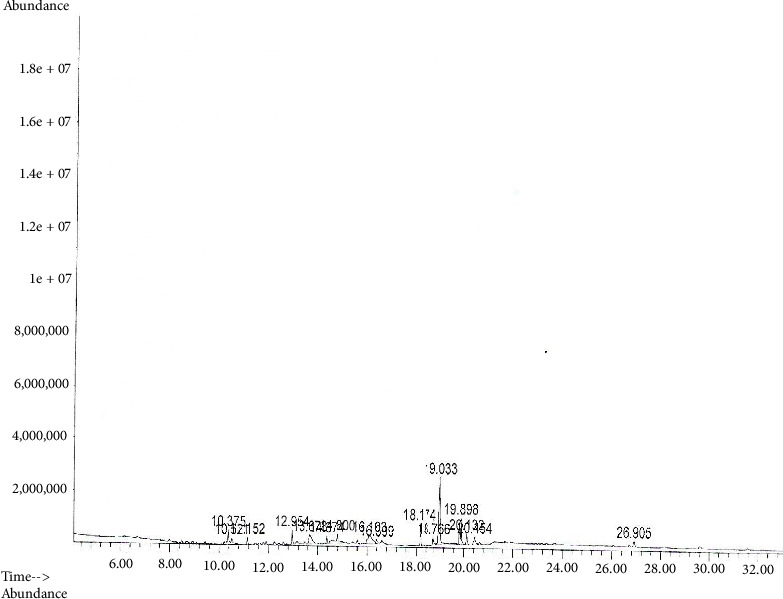
GC-MS chromatogram of MESh.

**Figure 2:**
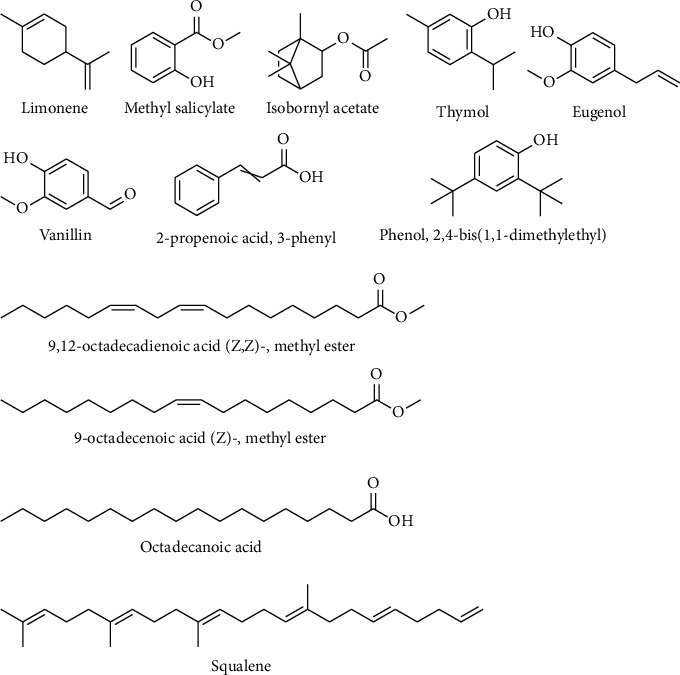
Chemical structures of phytochemicals identified in this study.

The GC-MS analysis of the extract identified more than 12 phytocompounds with various concentrations and retention times (Table 4; Figures 1 and 2). The detected phytocompounds belonged to different classes of phytocompounds, such as cyclic monoterpenes, salicylates, bicyclic monoterpenes, phenolic monoterpenes, polyphenols, fatty acids, and terpenes. The phytocompounds with the least concentration were squalene and limonene, while octadecanoic acid had the highest concentration (Table 4; Figures 1 and 2).

## 4. Discussion

An acute toxicity test assesses the adverse effects that occur after a single-dose exposure to a toxicant or test substance. This assay is usually conducted to provide insight into the possible toxicity of a novel molecule or medicine. It is mostly carried out in rodents. Animals are administered orally with the test material and then monitored for possible toxicity sign, changes of behaviors or fatalities for 14 days. Toxicological classification of toxicants begins with the LD_50_ value, or the amount required to kill 50% of the test animal [19].

In this study, the mice that received MESh at the single-dosage exposure of 150, 548, and 2000 mg/kg bw did not reveal any toxicity signs or behavioral changes, including fatalities, for 14 days, suggesting that the extract was nontoxic. The LD_50_ of the extract exceeded 2000 mg/kg bw in mice, implying that the extracts may be considered relatively safe on a single-dose exposure [26].

This study also found that MESh possesses an anti-inflammatory effect on formalin-induced edema in mice. Formalin induces inflammation through the production of histamine, bradykinin, serotonin, pro-inflammatory cytokines (such as interleukin (IL)-1β, IL-6, and tumor necrosis factor-alpha (TNF-α)), prostaglandin E2 (PGE2), and nitric oxide [27]. The mice that received the extract at 25, 50, and 100 mg/kg bw, as well as the reference drug, diclofenac, revealed a reduction in paw edema, suggesting an anti-inflammatory effect. Several research studies have documented antiedema activities of methanol plant extracts in mice. Yimer et al. [20] demonstrated that methanol extract of *Echinops kebericho* M. had an antiedema effect on formalin-induced edema in mice. Asefa et al. [22] also revealed that methanol extract of *Verbascum sinaiticum* Benth had an anti-inflammatory effect on formalin-induced edema in mice.

Nonsteroidal anti-inflammatory drugs (NSAIDs), such as diclofenac, are frequently used to treat inflammation [28]. The mechanism of action of diclofenac in the reduction of inflammation is through the inhibition of the synthesis of cyclooxygenase-2 (COX-2), an enzyme that promotes the synthesis of PGE2 [29]. The antiedema effect of the extract in this study may be attributed to the suppression in the production and release of PGE2 or inhibition of bradykinin, serotonin, histamine, pro-inflammatory cytokines (TNF-α, IL-1β, and IL-6), COX-2, and nitric oxide.

The antiedema effect of the extract was in a dose-dependent response, indicating that an increase in dosage resulted in increased bioavailability of bioactive components, as well as efficacy. Besides, the efficacy of the extract increased in the 3rd and 4th hours relative to the 1st^t^ and 2nd hours. This phenomenon may be explained by the gradual passive diffusion of bioactive components across the cellular membrane [30]. The antiedema effect of the extract was associated with phytocompounds that were detected using GC-MS.

Thymol has been documented to inhibit inflammatory mediators like TNF-α and IL-(1β and 6) [31]. Eugenol has been shown to inhibit the synthesis of inflammatory mediators such as induction of inducible nitric oxide synthase (iNOS), COX-2, PGE2, TNF-α, IL-1β, and IL-6, as well as regulation of the nuclear factor kappa B (NF-κB) pathway [28]. Vanillin has been established to regulate the NF-κB pathway, as well as inhibit the synthesis of COX-2 and induce iNOS [32]. Phenol, 2,4-bis(1,1-dimethylethyl), has been documented to inhibit the synthesis of inflammatory mediators such as TNF-α, IL-1β, IL-6, and COX-2 [33].

Limonene has been demonstrated to inhibit inflammatory mediators such as TNF-α, IL-1β, IL-6, iNOS, PGE2, and COX-2 [31, 34]. Methyl salicylate has been documented to inhibit IL-1β, IL-6, COX-2, and PGE2 synthesis, as well as regulation of the NF-κB pathway [35]. Squalene has been established to suppress the synthesis of inflammatory mediators such as TNF-α, IL-(1β and 6), iNOS, and COX-2, as well as the regulation of the NF-κB pathway [32, 33].

The current study also demonstrated that MESh had an antinociceptive effect in early and late phases following formalin-induced nociception in mice. Formalin induces pain through the release of plasma proteins (such as PGE2, histamine, bradykinin, and serotonin) and migration of leukocytes, mainly monocytes and neutrophils [36]. Furthermore, COX-2, which has nociceptive properties, is usually overexpressed in response to tissue injury [37]. The antinociceptive potential of the extract was in a dose-dependent manner, suggesting that an increase in the dosage resulted in an increased bioavailability of antinociceptive agents.

The findings of the current study correlate with previous studies. For instance, Kimuni et al. [23] documented that methanol extracts of *Lantana camara*, *Ocimum gratissimum*, and *Cissampelos pareira* revealed antinociceptive effects on formalin-induced pain in mice. In addition, Asefa et al. [22] reported that methanol extract of *V. sinaiticum* had an analgesic effect following formalin-induced pain in mice. Uddin et al. [38] also found that methanol extract of *Diospyros malabarica* had an analgesic effect against formalin-induced pain in mice.

The formalin test is a common in vivo screening method utilized to assess the analgesic effects of new analgesic agents in rodents. The rodents exhibit biphasic nociceptive behavioral reactions (early and late phases) of paw biting, licking, and lifting after formalin is injected into the paw's subplantar tissue [39]. The early phase represents acute peripheral pain, most likely as a result of nociceptors being directly activated through transient receptor potential A1 (TRPA1) channels. It is believed that sensitization of central nervous system neurons in the dorsal horn causes the late-phase nociceptive response and is a factor in neuropathic pain [40].

Diclofenac is highly prescribed to treat pain, and therefore, it was chosen as the reference drug in this study. Diclofenac inhibits the activity of COX-2, thereby suppressing the production of prostanoids like PGE2, thromboxanes, and prostacyclins, which are crucial components of the nociceptive response. Diclofenac downregulates sensitized peripheral pain receptors, and therefore it is responsible for peripheral analgesic effects. This appears to be achieved by activating adenosine triphosphate-sensitive potassium channels, which in turn stimulates the L-arginine nitric oxide cyclic guanosine monophosphate signaling pathway [41].

The extract was able to reduce the time spent on nociception in the early and late phases. This implies that the extract was involved in both peripheral and central antinociceptive activities. This is most likely due to the inhibition in the synthesis of bradykinin, histamine, serotonin, COX-2, and PGE2, as well as the inhibition of central mechanisms. The antinociceptive potential of the extract was associated with the secondary metabolites that were identified using GC-MS analysis.

Eugenol has been documented to inhibit the synthesis of COX-2 and PGE2 [34]. Eugenol has also been documented to ameliorate neuropathic pain [42]. Vanillin has been established to inhibit the production of COX-2 [32]. Limonene has been shown to inhibit the synthesis of PGE2 and COX-2 [43]. Methyl salicylate also has been documented to suppress COX-2 and PGE2 synthesis [35].

This study also established that MESh possesses an antipyretic effect on turpentine-induced pyrexia in Wistar rats. Fever was induced using turpentine, an exogenous pyrogen that stimulates endogenous pyrogens such as TNF-α, interferon-gamma (IFN-γ), IL-1β, and IL-6 to initiate the synthesis of PGE2. The buildup of PGE2 in the hypothalamic preoptic area modifies the thermoregulatory set point, elevating the body's core temperature [4].

The pyretic rats that received the extracts, as well as the reference drug, diclofenac, noted a reduction in rectal temperatures throughout the treatment period, suggesting an antipyretic effect. The reference drug diclofenac ameliorates elevated body temperatures through COX pathway inhibition, thus suppressing the synthesis of PGE2 [44]. It is postulated that the extract could have reduced rectal temperatures through inhibition of PGE2 synthesis or by blocking the synthesis of endogenous pyrogens like IL-6 and IL-1β [4]. Previous research studies have documented the antipyretic effects of methanol plant extracts on turpentine-induced pyrexia in rats. For instance, Nthiga et al. [24] found that methanol extract (stem bark) of *Landolphia buchananii* and *Harrisonia abyssinica* had antipyretic effects on turpentine-induced pyrexia in rats.

The extract exhibited a dose-dependent antipyretic effect. This could be explained by rapid metabolism and clearance, including inactivation of bioactive agents at the lower dosage compared to the higher dosage [25]. The antipyretic effect of the extract was also higher in the 3rd and 4th hours of the study compared to the 1st and 2nd hours. This could be ascribed to a steady passive diffusion of the active phytochemicals through the cell membrane, thus increasing the bioavailability of bioactive agents in the 3rd and 4th hours. It is also possible that some phytocompounds were biotransformed to bioactive compounds, thus conferring better antipyretic activity in the 3rd and 4th hours of the study [45]. The antipyretic effect of the extract could be attributed to the secondary metabolites (phytocompounds) that were identified using GC-MS in this study.

Eugenol has been shown to inhibit the production of endogenous pyrogens such as TNF-α, IL-1β, and IL-6, including PGE2, which is responsible for alteration in the thermoregulatory set point of the hypothalamus [28]. Methyl salicylate is often metabolized to salicylic acid, which has been documented to downregulate the synthesis of PGE2, IL-1β, and IL-6 [35].

This study also evaluated qualitative and quantitative phytochemical screening of MESh using GC-MS. The phytochemical analysis and chemotaxonomic research of medicinal plants with physiologically active components heavily rely on GC-MS analysis [46]. The GC-MS analysis uses the mass-to-charge ratio and retention time to separate, detect, and identify compounds. The separation of individual components in a mixture is achieved by gas chromatography, after which the separated compounds are ionized, detected using a photodetector, and identified using mass spectrometry based on their mass-to-charge ratio. The GC-MS technique identifies compounds that are nonpolar, volatile, and with lower molecular weight [40, 41].

The phytocompounds that were detected in this study belonged to various classes of phytocompounds, including cyclic monoterpenes, salicylates, bicyclic monoterpenes, phenolic monoterpenes, phenols, fatty acids, and terpenes. The antipyretic, antinociceptive, and anti-inflammatory activities that were reported in this study could be associated with the bioactive phytocompounds that were detected using GC-MS.

## 5. Conclusions

This study concluded that MESh possesses anti-inflammatory, antinociceptive, and antipyretic activities. The extract was also safe at a maximum single-dose exposure of 2000 mg/kg bw. Thus, the phytocompounds of MESh can be harnessed as a lead in developing new anti-inflammatory, antinociceptive, and antipyretic agents. This study suggests further studies on the mechanisms of action of the extract involved in the observed anti-inflammatory, antinociceptive, and antipyretic effects.

## Figures and Tables

**Table 1:** Anti-inflammatory effect of MESh on formalin-induced paw edema in mice.

Group	Percentage paw edema inhibition (mm)			
	1 h	2 h	3 h	4 h
Negative control	5.86 ± 0.43^aB^	6.17 ± 0.66^aAB^	8.92 ± 0.51^aA^	8.18 ± 0.52^aA^
	(4.02 ± 0.02)	(4.05 ± 0.03)	(4.13 ± 0.06)	(4.10 ± 0.05)

Normal control	0.00 ± 0.00^c^	0.00 ± 0.00^b^	0.00 ± 0.00^b^	0.00 ± 0.00^b^
	(2.68 ± 0.04)	(2.68 ± 0.04)	(2.68 ± 0.04)	(2.68 ± 0.04)

Positive control	0.92 ± 0.07^bA^	−4.90 ± 0.37^dC^	−7.03 ± 0.29^eCD^	−8.22 ± 0.19^eD^
	(3.76 ± 0.04)	(3.54 ± 0.05)	(3.46 ± 0.04)	(3.42 ± 0.04)

25 mg/kg bw	1.22 ± 0.16^bA^	−1.27 ± 0.16^cC^	−4.13 ± 0.23^cD^	−4.34 ± 0.15^cD^
	(3.82 ± 0.02)	(3.73 ± 0.02)	(3.62 ± 0.02)	(3.61 ± 0.02)

50 mg/kg bw	1.07 ± 0.14^bA^	−1.93 ± 0.10^cC^	−4.67 ± 0.18^cdD^	−6.13 ± 0.29^dD^
	(3.77 ± 0.05)	(3.65 ± 0.04)	(3.55 ± 0.04)	(3.50 ± 0.05)

100 mg/kg bw	0.59 ± 0.15^bcA^	−2.47 ± 0.23^cB^	−5.37 ± 0.23^dC^	−7.43 ± 0.42^eD^
	(3.74 ± 0.04)	(3.63 ± 0.04)	(3.52 ± 0.04)	(3.45 ± 0.05)

*Note:* Mean ± SEM with distinct lowercase superscript letters at each hour differed significantly (*p* < 0.05) using one-way ANOVA and Tukey's multiple comparisons. Mean ± SEM with distinct uppercase superscript letters along the row differed significantly (*p* < 0.05) using repeated measures ANOVA with Bonferroni-adjusted multiple comparisons. Mean ± SEM within parentheses represents paw diameter (mm).

**Table 2:** Analgesic effect of MESh on formalin-induced pain in mice.

Group	Percentage of pain inhibition (seconds)	
	Early phase	Late phase
Negative control	0.00 ± 0.00^e^	0.00 ± 0.00^f^
	(136.00 ± 4.49)	(155.00 ± 3.46)

Normal control	100.00 ± 0.00^a^	100.00 ± 0.00^a^
	(0.00 ± 0.00)	(0.00 ± 0.00)

Positive control	66.18 ± 0.84^b^	73.68 ± 0.88^b^
	(46.00 ± 1.14)	(40.80 ± 1.36)

25 mg/kg bw	41.91 ± 0.84^e^	45.16 ± 0.76^e^
	(79.00 ± 1.14)	(85.00 ± 1.18)

50 mg/kg bw	55.88 ± 0.93^d^	60.77 ± 0.66^d^
	(60.00 ± 1.26)	(60.80 ± 1.02)

100 mg/kg bw	61.18 ± 0.75^c^	66.71 ± 0.93^c^
	(52.80 ± 1.02)	(51.60 ± 1.44)

*Note:* Mean ± SEM with distinct superscript letters at each phase differed significantly (*p* < 0.05) using one-way ANOVA and Tukey's multiple comparisons. Mean ± SEM within parentheses represents time (seconds) of nociception.

**Table 3:** Antipyretic effect of MESh on turpentine-induced fever in rats.

Group	Percentage pyretic inhibition (°C)			
	1 h	2 h	3 h	4 h
Negative control	1.55 ± 0.19^aA^	2.49 ± 0.34^aA^	2.28 ± 0.27^aA^	1.97 ± 0.20^aA^
	(39.28 ± 0.06)	(39.64 ± 0.05)	(39.56 ± 0.04)	(39.44 ± 0.02)

Normal control	−0.01 ± 0.12^bA^	−0.01 ± 0.09^bA^	−0.01 ± 0.17^bA^	−0.11 ± 0.07^bA^
	(37.04 ± 0.08)	(37.04 ± 0.02)	(37.04 ± 0.07)	(37.00 ± 0.05)

Positive control	−0.88 ± 0.16^cA^	−2.53 ± 0.57^eB^	−3.05 ± 0.20^dC^	−3.31 ± 0.09^dC^
	(38.32 ± 0.07)	(37.68 ± 0.07)	(37.48 ± 0.07)	(37.38 ± 0.04)

25 mg/kg bw	−0.05 ± 0.21^bA^	−0.73 ± 0.10^bcB^	−1.61 ± 0.19^cBC^	−1.97 ± 0.13^cC^
	(38.52 ± 0.08)	(38.26 ± 0.09)	(37.92 ± 0.06)	(37.78 ± 0.05)

50 mg/kg bw	−0.26 ± 0.21^bA^	−1.09 ± 0.15^cdB^	−1.77 ± 0.17^cC^	−2.39 ± 0.17^cD^
	(38.34 ± 0.05)	(38.02 ± 0.05)	(37.76 ± 0.08)	(37.52 ± 0.07)

100 mg/kg bw	−0.57 ± 0.10^bcA^	−1.71 ± 0.11^deB^	−2.23 ± 0.16^cdBC^	−2.54 ± 0.17^cC^
	(38.32 ± 0.08)	(37.88 ± 0.07)	(37.68 ± 0.07)	(37.56 ± 0.03)

*Note:* Mean ± SEM with distinct lowercase superscript letters at each hour differed significantly (*p* < 0.05) using one-way ANOVA and Tukey's multiple comparisons. Mean ± SEM of distinct uppercase superscript letters along the row differed significantly (*p* < 0.05) using repeated measures ANOVA with Bonferroni-adjusted multiple comparisons. Mean ± SEM within parentheses represents temperatures in °C.

**Table 4:** GC-MS phytochemical profile of MESh.

RT (min)	Chemical name	Phytochemical class	Molecular formula	Conc. (μg/g)
6.68	Limonene	Cyclic monoterpene	C_10_H_16_	136.24
9.34	Methyl salicylate	Salicylate	C_8_H_8_O_3_	152.15
9.43	Isobornyl acetate	Bicyclic monoterpenes	C_12_H_20_O_2_	196.29
11.15	Thymol	Phenolic monoterpene	C_10_H_14_O	150.22
11.66	Eugenol	Polyphenol	C_10_H_12_O_2_	164.2
12.43	Vanillin	Polyphenol	C_8_H_8_O_3_	152.15
13.16	2-Propenoic acid, 3-phenyl	Fatty acid	C_9_H_8_O_2_	148.16
13.74	Phenol, 2,4-bis(1,1-dimethylethyl)	Polyphenol	C_14_H_22_O	206.33
19.89	9,12-Octadecadienoic acid (Z,Z)-, methyl ester	Fatty acid	C_19_H_34_O_2_	294.47
19.89	9-Octadecanoic acid (Z)-, methyl ester	Fatty acid	C_19_H_36_O_2_	296.49
20.65	Octadecanoic acid	Fatty acid	CH_3_(CH_2_)_16_COOH	328.49
26.91	Squalene	Triterpene	C_30_H_50_	136.24

## Data Availability

Data are openly available in a public repository that issues datasets with DOIs. Repository URL: https://data.mendeley.com/preview/hh4jspzf8f?a=8a56c3a5-4da0-4079-899a-773122d73742. Repository Name: Mendeley Data.
